# Embedding cultural competency and cultural humility in undergraduate pharmacist initial education and training: a qualitative exploration of pharmacy student perspectives

**DOI:** 10.1007/s11096-023-01665-y

**Published:** 2023-12-08

**Authors:** Anna Robinson-Barella, Christopher Takyi, Hayley K. Y. Chan, Wing Man Lau

**Affiliations:** 1 School of Pharmacy, King George VI Building, Newcastle University, Newcastle, NE1 7RU UK; 2 Population Health Sciences Institute, Newcastle University, Newcastle, UK; 3 School of Medicine, Faculty of Medical Sciences, Newcastle University, Newcastle, UK

**Keywords:** Culture, Cultural competency, Education, pharmacy, Pharmacists, Qualitative research

## Abstract

**Background:**

Emphasis has been placed upon embedding equity, diversity and inclusion within the initial education and training of healthcare professionals, like pharmacists. Yet, there remains limited understanding of how best to integrate cultural competency and cultural humility into undergraduate pharmacy student training.

**Aim:**

This qualitative study explored the views of pharmacy students to understand perspectives on, and identify recommendations for, embedding cultural competency and cultural humility within pharmacy education and training.

**Method:**

Undergraduate pharmacy students from one UK-based School of Pharmacy were invited to participate in an in-person, semi-structured interview to discuss cultural competency in the pharmacy curriculum. Interviews were conducted between November 2022 and February 2023 and were audio-recorded and transcribed verbatim. Reflexive thematic analysis enabled the development of themes. QSR NVivo (Version 12) facilitated data management. Ethical approval was obtained from the Newcastle University Ethics Committee.

**Results:**

Twelve undergraduate pharmacist students, across all years of undergraduate training, were interviewed. Three themes were developed from the data, centring on: (1) recognising and reflecting on cultural competency and cultural humility; (2) gaining exposure and growing in confidence; and (3) thinking forward as a culturally competent pharmacist of the future.

**Conclusion:**

These findings offer actionable recommendations to align with the updated Initial Education and Training standards from the United Kingdom (UK) pharmacy regulator, the General Pharmaceutical Council; specifically, *how* and *when* cultural competency teaching should be embedded within the undergraduate pharmacy curriculum. Future research should further explore teaching content, learning environments, and methods of assessing cultural competency.

**Supplementary Information:**

The online version contains supplementary material available at 10.1007/s11096-023-01665-y.

## Impact statements

- Person-centred care is underpinned by equity, diversity and inclusion, yet there remains limited understanding of how best to educate and train pharmacy students in this way.
- This qualitative work offers actionable recommendations to integrate cultural competency education within undergraduate pharmacy curricula.
- These results can be used to inform curriculum design, specifically *how* and *when* cultural competency training could be embedded in training.
- These findings align with the recently updated UK General Pharmaceutical Council standards for the Initial Education and Training for pharmacists.

## Introduction

With globalisation and immigration rates increasing in recent years, many countries have seen rapid changes to their population race-ethnic demographics [1–6]. Specifically within the United Kingdom (UK), recent 2021 census data has reported increases in the number people from ethnic minority communities, compared to previous years [7]. Increases in diversity on a population-level can bring challenges and opportunities for healthcare systems, providers and policymakers to provide care services that are culturally competent, particularly at an individual-level. Cultural competency refers to the ability of organisations and individual professionals to provide healthcare services which meet the social, linguistic and cultural needs of patients [8, 9], while cultural humility practices self-reflection and lifelong learning about another’s culture as well as their own [10]. Evidence has demonstrated that healthcare systems which can deliver culturally competent services can help reduce ethnic inequalities, whilst simultaneously improving health outcomes and quality of care [8, 11–13]. Historically, a lack of cultural competency and cultural humility shown by healthcare providers or systems has led to higher levels of patient dissatisfaction [14], poorer rates of access to and attendance at clinics [15, 16] and, detrimentally, worse health-related outcomes for underrepresented and minoritised communities [17].

The profession of pharmacy has been recognised as a key player in the provision of accessible and person-centred healthcare, globally [18–20]. Wide-ranging research has identified improvements in person-centred care when pharmacists develop and practice with culturally competent attitude, knowledge and skill [21–26]. A recent scoping review explored the acceptability and effectiveness of pharmacist-led health services for minoritised populations [27], and key recommendations to provide culturally appropriate pharmacy-based services for ethnic minority communities have been identified [23].

In December 2020, the United Kingdom (UK) General Pharmaceutical Council (GPhC) approved and published new standards for the initial education and training of undergraduate pharmacy students where there was a ‘greater emphasis on equality, diversity and inclusion’ [28]. However, there still remains limited understanding of the best way to educate, prepare and support pharmacists (and members of the pharmacy team) to deliver healthcare with cultural competency and cultural humility. Specifically, there is a lack of clarity into curriculm design and content to best support undergraduate student pharmacists in their initial education and training.

### Aim

This qualitative study explored the views of pharmacy students to understand perspectives on, and identify recommendations for, embedding cultural competency and cultural humility within pharmacy education and training.

### Ethics approval

Ethical approval was obtained from the Newcastle University Ethics Committee (reference: 25,004/2022, date granted October 2022) and research governance was followed in accordance to university research policies.

## Method

### Recruitment and sampling

This study has been reported according to the COnsolidated criteria for REporting Qualitative research (COREQ) checklist. Recruitment was facilitated by two researchers (CT and HKYC) via email invitations; emails were sent out to the whole pharmacy student cohort at the time of the study taking place (n = 500). In total, 12 participants expressed interest and were subsequently emailed a participant information sheet and consent form detailing the research purpose and aim; participants who gave their written consent were enrolled in the study. There was no relationship established between the researchers and the participants before study commencement or recruitment. Inclusion criteria comprised: current pharmacy students at Newcastle University School of Pharmacy in the UK; students were recruited across all four years of their degree. Purposive sampling ensured (i) representation in views across all stages of the four-year degree and (ii) diversity within the population on accounts of participant ethnicity, age and pharmacy work-experience outside of university.

### Semi-structured interviews

In-depth, semi-structured interviews were conducted by one researcher (CT, a male medical student with experience of qualitative research) between November 2022 and February 2023. All interviews were conducted in-person, either as one-to-one or in pairs at their request (Table 1); this approach was taken to support participants to feel comfortable and confident, given it was their first time being involved in a research project. A semi-structured topic guide (Supplementary File 1) was developed based on three pilot interviews and existing literature [23, 24, 29].

**Table 1:** Participant demographics

Participant number	Self-reported sex	Age range (years)	Stage (year) of study	Self-reported ethnicity	History of pharmacy work experience? (Yes / No)	Semi-structured interview format (Individual or Pair)	Interview duration (minutes, seconds)
1	F	< 20	1	Black, British	N	I	42m 38s
2	M	20–25	2	White, British	Y	I	20m 19s
3	F	25 +	3	Latin American	Y	I	22m 07s
4	M	20–25	3	Black, African	Y	I	26m 46s
5	F	20–25	4	White, British	Y	P	41m 39s
6	F	20–25	4	White, British	Y	P	
7	M	< 20	2	White, Polish	Y	P	52m 22s
8	M	< 20	2	Mixed race, American	N	P	
9	F	20–25	3	Asian, Pakistani	N	I	32m 22s
10	F	20–25	3	White, Canadian	Y	I	23m 21s
11	F	25 +	4	White, British	Y	I	39m 52s
12	F	< 20	2	Asian, Pakistani	N	I	22m 41s

### Data analysis

All interviews were audio-recorded to enable data analysis. The audio files were encrypted and transcribed verbatim by CT. All interview data were anonymised at the point of transcription. All transcripts were checked for accuracy by HKYC and AR-B; immediately following this, the files were deleted. Participants did not provide comment on the transcripts nor feedback on the results.

Following reflexive thematic analysis [30, 31], the principle of constant comparison guided an iterative process of simultaneous data collection and data analysis. Close and detailed reading of the transcripts enabled data familiarisation and inductive coding processes. Initial descriptive codes were identified in a systematic manner across the data sets—these were then sorted into common coding patterns, which enabled the development of analytic themes. The themes were reviewed, refined and named once coherent and distinctive. Two authors performed reflexive data analysis through discussion (CT and AR-B) and if agreement was not reached, they sought consensus from the wider research team (HKYC and WML). Post-interview field notes enhanced the reflective process and enabled iterative and inductive working. QSR NVivo (version 12) software facilitated data management. The research team were in agreement that data sufficiency and information power occurred after conducting eight interviews with a total of 12 participants [32, 33].

### Considerations when reporting participant demographics

Collecting data on a person’s ethnic group is complex, since ethnicity in itself is a multi-faceted and changing phenomenon. The UK Office of National Statistics ‘Ethnic group, national identity and religion’ [7] and the National Institutes of Health (NIH) ‘Racial and Ethnic Categories and Definitions for NIH Diversity Programs and Other Reporting Purposes’ [34] guides were used to inform the reporting of participant ethnicity for this study (as demonstrated in Table 1).

## Results

### Participant demographics

In total, 12 participants were recruited and interviewed for this study (Table 1). All participants were pharmacy undergraduate students studying at one School of Pharmacy in the UK. Eight interviews were conducted as individuals and two interviews were conducted in pairs. Considering their pharmacy education level, one participant was in their first year of study (9%), four were in their second year (33%), four were in their third year (33%) and three were in their fourth year (25%). Participant self-reported ethnicities included a range of minority and majority groups, and included the views of five international students. There were no refusals to partake, participant drop outs or repeat interviews.

**Fig. 1:**
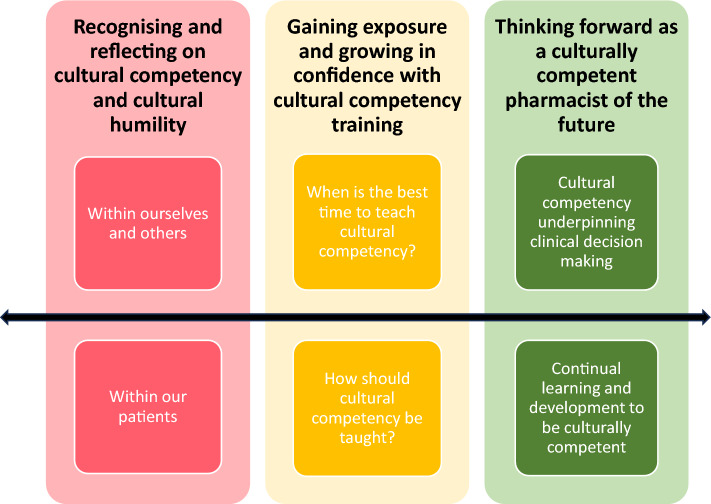
The three themes and sub-themes developed in this study which appear fundamental in shaping the initial education and training of pharmacy students underpinned by cultural competency and cultural humility. The double-ended arrow and choice of colours indicate the iterative personal development and education in the space of cultural competency –the red colour represents processes of recognition and self-reflection; the amber colour indicates strategies to promote growth in confidence; and green denotes the forward thinking approaches of a culturally competent pharmacist when practicing in the future

Three overarching themes were developed to reflect the perceptions of pharmacy students on the integration of cultural competency and cultural humility within pharmacy education: (1) recognising and reflecting on cultural competency and cultural humility; (2) gaining exposure and growing in confidence with cultural competency training; and (3) thinking forward as a culturally competent pharmacist of the future (Fig. 1). The three themes, and their sub-themes, are discussed in turn (Table 2 contains further quotes).

**Table 2:** Additional verbatim quotes included to illustrate the themes and sub-themes of this work

Theme	Sub-theme	Additional illustrative, verbatim quotes
Recognising and reflecting on cultural competency and cultural humility	Within ourselves and in our student cohort	*“Having a good awareness of all the different types of people or beliefs you could come across in any sort of relationship, like not just patient to healthcare professional, but like your classmates or your friends too”* (Participant 11, Stage 4)
	Within our patients	*“Being able to tailor yourself and the care you’re providing to the individual in front of you, rather than it being the same blanket approach for everyone”* (Participant 6, Stage 4)
Gaining exposure and growing in confidence with cultural competency	When is the best time to teach cultural competency?	*“Ideally we (students) should have that mindset from an early stage… something that’s at the front of your mind so you carry it with you”* (Participant 4, Stage 3) *“When I was in first year, I probably just needed to know what it (cultural competency) meant and that it’s something we’re going to touch on all the way through (the degree)”* (Participant 11, Stage 4), *“In real life once we qualify, we’re not going to have someone with only one isolated condition… and they’re not going to fit exactly into one ‘box’… that’s not reality! (laughs) so, in the (cultural competency) teaching on the degree, I think it should take the same approach and feature in a bit of everything, all the way through”* (Participant 6, Stage 4) *“Gradual introductions to what cultural competency is right when you’re just starting out as a pharmacy student”* (Participant 8, Stage 2)
	How should cultural competency be taught?	*“Seminar classes, where it’s a smaller group, and you could discuss through a patient case – that’s where it (cultural competency teaching) would work well I think, because you’re in a small, safe space where you can ask questions and expand your thinking”* (Participant 3, Stage 3) *“Where you have a mock patient, follow them through, discuss through their diagnoses, their meds (medication) and then think about the holistic lifestyle impacts, which is where culture (and cultural competency) comes into play”* (Participant 9, Stage 3)
Thinking forward as a culturally competent pharmacist-of-the-future	Cultural competency underpinning clinical decision making	*“When you’re meeting real people and seeing it first hand… whether it be an adult patient, or a child in the paediatric unit, it makes it all real what you’re learning about … and you get why this is all important”* (Participant 5, Stage 4)
	Continual learning and development to be culturally competent	*“Cultural competency does not just finish in first year or something like that… it has to continue throughout your career as a pharmacist”* (Participant 8, Stage 2) *“Preparing me for my future as a pharmacist who doesn’t discriminate and who has a culturally open mind”* (Participant 11, Stage 4) *“If there was like a mandatory CPD on anything new coming out, or just even if it was a refresher course (as part of foundation training year) covering like ‘how to speak to people form different religious beliefs about medicines’ … or like seeing it included in the prescribing course or the clinical diploma … it shouldn’t just end at university”* (Participant 5, Stage 4)

### Theme 1: Recognising and reflecting on cultural competency and cultural humility

#### Within ourselves and in our student cohort

Many participants began by reflecting on what they knew and understood cultural competency to be. Two students described understanding cultural competency as being centred in *awareness*. One student in their second year of pharmacy education described

“being culturally competent… I feel like it’s mostly just being aware of like, differences, across different people and cultures” (Participant 2, Stage 2).

One student shared how their upbringing has shaped their appreciation for diversity within people and places, describing

“where I lived growing up, it was in a really multi-cultural place with people of all ethnicities in my school, class, friendship groups… that’s just normal to me – to me, cultural competency is probably viewing society and others in a non-judgemental way… but maybe that’s not the same for everyone” (Participant 1, Stage 1).

Another student reflected only becoming aware of wider diversity and cultures

“only really when I moved to University and began to mix with more wider diverse people and started learning more about other ways of doing things” (Participant 2, Stage 2).

“I can see why it (cultural competency) is important because it’s encouraging you to open your eyes and mind more and if we’re going to be pharmacists, we need to show we care about other people’s beliefs” (Participant 2, Stage 2).

#### Within our patients

Students also demonstrated reflections that extended beyond themselves, to the patient population they would be providing care for. One final year student described culturally competent approaches as those which identify each patient as

“unique and treating them as such”, instead of adopting a style of *“a one-size-fits-all”* (Participant 6, Stage 4).

Many students noted that cultural competency and cultural humility extended beyond ethnicity as a single entity. Instead, students discussed considerations that take

“into account people’s culture—there’s aspects of religion, race, ethnicity, sexual orientation, the list can go on… it’s important you factor in the whole person” (Participant 5, Stage 4).

Students illuded to the importance of learning about wider issues that may also impact a person in receipt of healthcare, such as their sexual orientation.

“As much as it (cultural competency training) should be learning about like race and ethnicity factors, we cannot forget about learning about other factors that like, make up a person, like if they are gay or transgender that we’re treating” (Participant 3, Stage 3).

Two students made the connection between cultural competency and the General Pharmaceutical Council (GPhC) standards for pharmacy professionals, particularly in relation to person-centred care. One student stated

“cultural competency – it’s important for us to learn about because it also adheres to the GPhC’s standards, like we’re actually showing that we care about the person’s beliefs and they don’t feel just like a case to be solved” (Participant 12, Stage 2).

### Theme 2: Gaining exposure and growing in confidence with cultural competency training

#### When is the best time to teach cultural competency?

One of the main points of discussion in interviews was the need to grow student confidence, specifically by gaining exposure to issues exploring cultural competency within their education. Students suggested a number of ways of when cultural competency and cultural humility should feature within their training to gain this required exposure. One such suggestion centred around taught materials “*like specific seminar classes*” which were aimed at

“introducing the concept at the very start (of the undergraduate degree) so you’re made aware of what it is at a basic level” (Participant 3, Stage 3).

“When you start learning more clinical things, like from stage 2 onwards, and when it gets more complicated in stage 3 and 4, you need to be bringing the cultural focus back to everything – so if you’ve already covered it from stage 1, then it should become second nature” (Participant 5, Stage 4).

All student participants commented that the teaching of cultural competency should continue throughout the degree. But rather than taking place in standalone session(s), students felt that content should be embedded throughout all aspects of the degree. One student described

“rather than it being titled ‘this is your cultural competency teaching’ … I think it would be actually better if it featured a little bit in everything we do” (Participant 11, Stage 4).

Another student recognised the reality of the diverse people they will be treating once qualified, citing this as the reason to weave cultural competency education throughout (Table 2).

#### How should cultural competency be taught?

Recommendations were also made around the approaches and teaching styles that may be best suited for embedding cultural competency training within the current curriculum. Many participants drew connections between the learning environments that may be better suited for embedding cultural competency training within the current curriculum. Seminar classes, or classes that were smaller in size and more interactive than lectures, were deemed an ideal environment for cultural competency to be visited, and re-visited. Teaching that was linked to a patient case and where it echoed a real-life scenario was viewed as the most desirable (Table 2). 

There was a connection linking cultural competency education with supporting the knowledge, as well as confidence, of pharmacy students. One second year student spoke of wanting

“to feel more confident in the language I use or terms I use when I’m talking about this (cultural competency) so, to be honest, I would prefer it (teaching) to be in a smaller group so I’m not… embarrassed if I got it wrong” (Participant 2, Stage 2).

A first year student indicated their readiness to learn, but also the need for confidence, as they discussed wanting

“to just be able to learn, and have the knowledge, and have the confidence really to conduct a review with anyone, any type of person” (Participant 1, Stage 1).

A final year student reflected upon the development in their confidence and communication over time, recognising

“I was not confident about what words to even use at the start, but now, like there’s definitely been progress and I do feel more confident in thinking that way (with cultural competency)” (Participant 11, Stage 4).

It appeared that laying the foundations at the start of the degree, and building upon them throughout their education, was a desirable approach to take.

Other examples of opportunities to learn and develop cultural competency skills included experiential learning, specifically on placement. Many students, across all years of study, described the patient contact time they experienced on multi-sector pharmacy placements (a standard learning experience within their degree) as a key opportunity to put their learning into practice. Students discussed the value and benefit of hearing first-hand the lived-experiences of

“the actual people experiencing this… they should be the ones feeding back and keeping us right (around issues relating to cultural competency) as they’re living it” (Participant 5, Stage 4).

### Theme 3: Thinking forward as a culturally competent pharmacist-of-the-future

#### Cultural competency underpinning clinical decision making

Thinking forward on their educational trajectory to becoming pharmacists, students acknowledged and shared examples of where cultural competency training can support them in clinical decision-making alongside patients. Students across stages two, three and four appreciated the importance of tailoring care to an individuals’ needs but they also extended this to encompass *culture* in the examples they provided. Many of these examples related to supporting the safe and effective use of medicines and, in particular, involved a patient from a minoritised community (see Table 2).

“Doing something in reality that I’d learnt about in the classroom ... a man of South Asian descent came to me on placement asking me about his medicines, well, the fact he wants to miss taking his medicines when it’s Ramadan ... I was able to help him because of what we’d covered in seminars” (Participant 11, Stage 4).

Second year students also eluded to the importance of appreciating person-centred ethnic considerations when diagnosing and treating disease; they discussed

“in hypertension, there’s certain ethnic groups that you can’t give ACE-inhibitors to … that’s really important for us to know to make sure we’re keeping patients safe” (Participant 7, Stage 3).

Another indicated the importance of including diversity within teaching materials, given that some dermatology conditions have

“some physical signs that you can see, but on certain skin colours, you can’t see the effects or it might look totally different” (Participant 8, Stage 2).

Students also acknowledged that cultural competency teaching can extend beyond the discipline of pharmacy practice. One student described how culturally competent approaches in the specialty of pharmaceutics has supported their knowledge of excipient suitability, given that

“certain religions can’t have certain ingredients, meaning certain medications are unsuitable … like those that have meat products in, or alcohol content” (Participant 8, Stage 2).

#### Continual learning and development to be culturally competent

Students also acknowledged that there is, and will always be, need for them to practice ongoing, continual personal development. When considering their education and training, students were mindful of the fast-paced changes and updates relating to cultural competency; one student discussed

“the terminology is changing all of the time, now even ‘BAME’, as a term, that was used loads fairly recently but even now it’s fading” (Participant 1, Stage 1).

“(Being a culturally competent pharmacist), it’s definitely going to be an ongoing process, we can’t know everything, but we can try to learn as much as possible because all patients need us to approach them sensitively” (Participant 11, Stage 4).

Developing cultural competency skills outside of the profession and transferring them into their role as a future pharmacist was also discussed. Students discussed

“independent learning that I could do myself, like reading up on the news and social media about how people are speaking up for trans rights… that can all feed back into my job and patients I’m seeing” (Participant 3, Stage 3).

Many students described reflective approaches that could be integrated into their personal and professional development. One student considered how

“writing reflective pieces on being in the scenario where cultural competency is required… it should be done as reflecting improves who you are as a pharmacist” (Participant 8, Stage 2).

A final year student considered ways that their pharmacy postgraduate education could continue the links established at an undergraduate level, including focus on cultural competency (Table 2).

## Discussion

### Statement of key findings

The findings of this work contribute to the growing evidence base of establishing culturally competent care provision within the pharmacy profession [23, 24, 29]. Uniquely, this study focuses on the initial education and training of pharmacy students and offers early insight to begin informing curriculum design, addressing the recently updated UK General Pharmaceutical Council standards around equality, diversity and inclusivity [28, 35]. Specifically, the authors sought to gather perspectives of current pharmacy students and explore associations between their perceptions of cultural competency and its value in both the pharmacy profession and wider healthcare settings.

### Strengths and weaknesses

This study captured the perspectives of current UK pharmacy students, representating views across all four stages of the degree (reflecting the typical duration of a UK-based Masters of Pharmacy undergraduate education). Efforts were made to ensure diversity within the sample regarding students’ reported ethnicity and previous experience of working within the profession (both of which may influence perception of cultural competency through lived-experience and/or in-practice). Furthermore, diversity was embodied throughout the entire research study, from topic conceptualisation, to the inclusion of ethnicity champions within the wider research team (HKG, LS and NA), to authors with experience across medicine (CT), pharmacy (HKYC, WML) and cultural inequity (AR-B). The research team acknowledge there were also some limitations to this study; while efforts were taken to ensure equal sex/gender representation amongst the participants, the majority of voices included in this work were female (n = eight). Furthermore, this study included students from one School of Pharmacy in the UK thus, findings may not be representative across other UK institutions and/or internationally within the education of a global healthcare profession.

### Interpretation

The value of embedding cultural competency training throughout all stages of their undergraduate degree echoes views shared by pharmacy profesionals and medical educators, who recognised mapping across all educational content in undergraduate training as vital [26, 36]. These findings echo those recognised internationally in medical education research conducted in the United Kingdom and Jordan [37] and the United States [38], as well as pharmacy education research from Australia [39] and Qatar [40]. Integrating cultural competency and cultural humility teaching alongside student self-reflection and self-assessment has been recommended as a way to educate medical students as culturally-sensitive healthcare professionals [41]. Whilst studies have started to address this in wider, global healthcare, there still remain gaps in knowledge about the optimal way to achieve a culturally competent curriculum within pharmacy training.

Pharmacy students, across all years of the undergraduate degree, perceived placement experience as valuable a opportunity for them to develop their skills and confidence with cultural competency. Similar findings have been reported previously in the international literature around the integration of situated placements to promote cultural competency in healthcare professional student education. Medical and nursing professions, thus far, are responsible for the majority of recent cultural competency education research [42–45], as well as it being recognised as a topic of interest amongst other professions such as occupational therapy [46, 47], school psychology [48] and rehabilitation counselling [49]. Kassam et al*.* addressed cultural competency in pharmacy education in Canada through an international service learning and community engagement scheme, where students reported increased knowledge and awareness of ethnic-centred health beliefs and health practices [50]. Further pharmacy-focused research, to better understand placement experiences as an opportunity for cultural competency development, is still much-needed.

Cultural competency was regarded as extending to topics beyond ethnicity-related inequity [51, 52]. In particular, education about marginalised groups, including those who are lesbian, gay, bisexual and transgender (LGBT +) was recognised as a vital component of cultural competency training [53]. In acknowledgement of the GPhC emphasis on equality, diversity and inclusion report, the Pharmacist Defence Association launched an action plan to support delivery of LGBT + inclusion in the UK MPharm programme [54] however, further evaluation and interrogation of curricula is needed [55, 56].

Students in this study recognised the need to not only include cultural competency and cultural humility training within their undergraduate degree, but also throughout their further postgraduate education. In 2021, the Centre for Pharmacy Postgraduate Education launched a learning campaign on ‘Culturally Competent Person-Centred Care’, designed for pharmacy professionals [57]. At present, research has yet to evaluate the impact of uptake of this training amongst postgraduate, qualified pharmacists; future studies may seek to address this to close the gap between pharmacist training at undergraduate and postgraduate levels [26].

### Further research

The specifics of teaching content, learning environments, placement opportunities, and even methods of assessing cultural competency that would be best suited to support the initial education and training of culturally competent pharmacy professionals, remains unknown. A recent systematic review identified heterogeneity in the current methods used to teach and assess cultural competency across pharmacy education, globally [58]; thus, future research should seek to unpick these elements further, whilst also recognising possible underpinning theoretical models or frameworks which may lead to successful cultural competency training [59]. In a bid to achieve an inclusive and diverse pharmacy curriculum, future research should seek to explore the integration of wider elements of cultural competency and humility within other seldom-heard, minoritised or marginalised groups.

## Conclusion

This qualitative study explored the perceptions of twelve student pharmacists relating to the integration of cultural competency teaching within undergraduate education. Data analysis developed overarching themes that identified: (i) where pharmacy curricula could support students to gain exposure and confidence with cultural competency and cultural humility education, as well as (ii) promote forward thinking by underpinning this learning in their practice as culturally competent pharmacists of the future. Recommendations have been made that centre on *how* and *when* cultural competency teaching could be embedded within the undergraduate pharmacy curriculum; however, future research should seek to explore specifics of teaching content, learning environments, and methods of assessment that would be best suited to support the initial education and training of culturally competent pharmacy professionals.

### Supplementary Information

Below is the link to the electronic supplementary material.

Supplementary file 1 (DOCX 19 KB)
