# Supplementary material for: Embedding cultural competency and cultural humility in undergraduate pharmacist initial education and training: a qualitative exploration of pharmacy student perspectives
Source: Int J Clin Pharm. 2023 Dec 8;46(1):166–76. doi: 10.1007/s11096-023-01665-y (PMC10830727; doi:10.1007/s11096-023-01665-y)
Supplement: Supplementary file 1 — (DOCX 19 KB) [file 11096_2023_1665_MOESM1_ESM.docx]

**Supplementary material 1:**

**Semi-structured interview topic guide**

The semi-structured interview questions were based around the following topic areas:

1. (Broader) Understanding of cultural competence/cultural humility in general
2. (Broad) Experiences of cultural competency within their personal life / place of work
3. (Narrowing) Experiences of engaging with cultural competency/cultural humility
4. (Narrow) Understanding of cultural competence in healthcare / in pharmacy
5. (Focused) Examples of cultural competence teaching within pharmacy education
6. (Focused) Training around cultural competence – current / previous / future
7. (Focused) Suggestions on supporting education on cultural competence
